# Association between Glucose-6-Phosphate Dehydrogenase Deficiency and Asthma

**DOI:** 10.3390/jcm10235639

**Published:** 2021-11-29

**Authors:** Alessandro Fois, Maria Pina Dore, Andrea Manca, Valentina Scano, Pietro Pirina, Giovanni Mario Pes

**Affiliations:** 1 Dipartimento di Scienze Mediche, Chirurgiche e Sperimentali, University of Sassari, Viale San Pietro 43, 07100 Sassari, Italy; agfois@uniss.it (A.F.); mpdore@uniss.it (M.P.D.); amanca89@tiscali.it (A.M.); valescano93@gmail.com (V.S.); pirina@uniss.it (P.P.); 2 Baylor College of Medicine, One Baylor Plaza Blvd., Houston, TX 77030, USA; 3 Sardinia Longevity Blue Zone Observatory, 08040 Ogliastra, Italy

**Keywords:** asthma, glucose-6-phosphate dehydrogenase deficiency, antioxidant defense, Sardinia

## Abstract

Background: Among the determinants contributing to the pathogenesis of asthma, antioxidant genetic factors play a leading role. Glucose-6-phosphate dehydrogenase (G6PD) is an enzyme that is competent to detoxify free radicals. Although a relationship between G6PD deficiency and asthma has been previously reported, the literature is still scanty. In this study, we test this hypothesis in a large cohort of patients from Sardinia, Italy. Methods: A retrospective case–control study was performed using data from 11,829 clinical records of outpatients referred to a teaching hospital for a medical visit. In total, 455 cases (asthma-positive) and 11,374 controls (asthma-negative) were compared for G6PD status using multivariable analysis, adjusting for all covariates. Results: Overall, G6PD deficiency was detected in 11.2% of study participants and was associated with an increased risk of asthma (odds ratio (OR) 1.63; 95% confidence interval (CI) 1.27–2.10). Additional variables significantly associated with asthma were female sex (OR 1.66; 95% CI 1.34–2.06), overweight/obesity (OR 1.56; 95% CI 1.27–1.92), smoking (OR 1.44; 95% CI 1.449–3.963), and high socioeconomic status (OR 1.40; 95% CI 1.16–1.70), whereas age was inversely related with asthma (OR 0.49; 95% CI 0.39–0.61). Conclusions: Our study shows that G6PD deficiency is an independent risk for asthma. These findings suggest that G6PD should be assessed in asthmatic patients for better risk stratification.

## 1. Introduction

Asthma is an inflammatory non-communicable disease of the small airways that affects more than 330 million people worldwide [1]. The prevalence rate shows differences between countries and studies [2], ranging from 0.2% in China to 21.0% in Australia [3]. Although asthma is considered the most common chronic disease among children, it can affect adults as well. As with other inflammatory diseases, asthma is more prevalent in adult females compared to males, although the gender disparity is reversed in childhood, where asthma is more frequent among boys than among girls [4]. The physiopathology of asthma is characterized by the persistent inflammation of small airways with eosinophilic infiltration in the mucosal lamina propria, leading to airway hyperresponsiveness to a wide variety of exogenous and endogenous stimuli [5]. Consequently, patients suffer from wheezing and cough, sometimes undergoing a progressive lifetime disability.

Apart from mucosal eosinophils, additional immune cells participate in long-lasting disease as well. Exposure to allergens triggers the production of specific IgE antibodies, leading to the overexpression of Th2 type T-cell response. The IgE bound to high-affinity receptors on dendritic cells facilitate allergen internalization; once inside the dendritic cell, the processing of allergens by cathepsin S and the subsequent selection of peptides loaded onto and presented by HLA molecules (MHC class II) are fundamental steps for these cells to act as antigen-presenting cells to T lymphocytes [6]. Presentation of a selected antigen peptide to the T-cell receptor induces sensitization and a subsequent immune response to the specific allergen [7].

Among the risk factors involved in the development of asthma, dietary, environmental, and genetic factors that are capable of reducing cell antioxidant capacity by increasing tissue vulnerability to oxidative stress raise the disease risk. For instance, selenium deficiency lowers red cell glutathione peroxidase activity and is associated with increased asthma risk [8]. Similarly, low dietary intakes of vitamins C and E appear to increase the risk as well [9,10]. Additional predisposing factors are family history, perinatal factors such as maternal smoking [11], age, diet and vitamin D deficiency [12], presence of other atopic diseases, maternal and childhood exposure to medications such as acetaminophen [13], proton pump inhibitors [14], certain antibiotics, and exposure to bacteria and bacterial products early in life. Moreover, abdominal obesity has been suggested to raise the risk of developing asthma [15]. The major environmental risk factor is a smoking habit, which seems to alter mucosal proteins [16]. Although the pathogenesis of asthma remains elusive, several genetic factors can interact with environmental risk factors to enhance the inflammation and tissue damage caused by oxidative stress, promoting the disorder.

Interestingly, several years ago, a few studies reported an increased risk for developing asthma in subjects carrying a glucose-6-phosphate dehydrogenase (G6PD) defect [17,18]. However, for several decades, the topic was shelved, and, until now, the literature on this association is scarce.

G6PD is known as the rate-limiting enzyme of the pentose phosphate pathway [19]. The enzyme produces nicotinamide adenine dinucleotide phosphate (NADPH), the essential cofactor in oxidoreductive metabolism, maintaining a high ratio of reduced/oxidized glutathione (GSH) and acting as a substrate for NADPH oxidase (NOX) and nitric oxide synthase (NOS). G6PD deficiency is the most common enzyme defect worldwide, affecting 400–500 million people [20]. Individuals harboring enzyme deficiency are generally asymptomatic but, in certain circumstances, may show episodic anemia. Upon exposure to fava beans, infections, or medicines with a high redox potential, including non-steroidal anti-inflammatory drugs, older red cells, where the G6PD deficiency gradient is higher, undergo hemolysis. Moreover, in newborns with G6PD deficiency, decreased bilirubin excretion may result in jaundice. More recently, it has been reported that G6PD deficiency may exert a proinflammatory effect depending on the animal or human model and the tissue involved [21].

On the Sardinian island in Italy, G6PD deficiency is very common, e.g., around 10–12% of the general population [22]. On the other hand, data according to the Gender Environment Interactions in Respiratory Diseases (GEIRD) indicate a relative risk of asthma in Italy of 6.6 (95% CI 6.1–7.1) and, more specifically, for Sassari (northern Sardinia), a relative risk of 7.3 (95% CI 6.0–8.8) [23]. An additional epidemiological study in southern Italy, including Sardinia and Sicily, reported a prevalence of asthma ranging between 2.6% and 3.2%, making the island the ideal model to test the G6PD/asthma association [24].

Based on these premises, we attempted to evaluate the association between asthma and G6PD deficiency in a defined population of northern Sardinia.

## 2. Materials and Methods

### 2.1. Study Design

This was a retrospective case–control, single-center study that took advantage of the availability of the clinical records of adult outpatients referred to a teaching hospital of northern Sardinia (Department of Internal Medicine, University of Sassari), Italy, from January 2002 to December 2019. Patients were referred by their family physicians and/or specialists for any reason. Patients with asthma were considered, and the controls were patients without asthma.

### 2.2. Eligibility Criteria

Records of personal information such as sex, age, smoking habits, socioeconomic status, anthropometric parameters (body height and weight), all signs and symptoms, treatments, and an accurate medical history were considered eligible for the analysis. More specifically, for the purpose of the study, the presence of asthma was retrieved from the computerized database among previously diagnosed conditions. Moreover, the diagnosis of asthma retrieved from patient’s chart was double-checked by matching the treatment (for example, inhaled corticosteroid or mixed inhaled corticosteroid, long-acting β2-agonist on maintenance or on demand, or short-acting β2-agonist upon need), according to the Global Initiative for Asthma (GINA) consensus [25]. Availability of G6PD status was considered the major inclusion criteria. Data from each patient were collected using a standard form for the entire study period, and each visit was supervised by the same attending physician.

### 2.3. Exclusion Criteria

Incomplete records or records belonging to patients younger than 18 years were not included in the analysis. In the case of multiple visits for the same patient within the given time period, only the most recent was considered for the analysis.

### 2.4. Diagnostic Criteria

G6PD status. Since Sardinia is a high-prevalence region for G6PD deficiency, the subjects are routinely assessed for G6PD status, especially before exposure to certain medications. The G6PD activity of whole blood was measured using a standard method, as previously described [26,27]. Less than 10% residual activity was used to define the presence of G6PD total deficiency, while residual activity ranging from 10% to 50% was used to define partial deficiency. Molecular analysis was not available for G6PD-deficient patients.

Asthma diagnosis. Asthma is a chronic obstructive condition characterized by both the inflammatory component and airway obstruction, which may be spontaneous or induced by different triggers, different from chronic obstructive pulmonary disease (COPD), which typically shows a fixed bronchial obstruction. The measure of pulmonary function assessed by spirometry is considered the gold standard to diagnose asthma [28]. According to the European Respiratory Society and American Thoracic Society (ERS/ATS) guidelines, a ratio between forced expiratory volume in the first second (FEV_1_) and forced vital capacity (FVC) less than 0.7 is the cut-off used to diagnose an obstructive respiratory pattern, while the GINA consensus suggests a cut-off of 0.8 [25]. To establish if the bronchial obstruction is permanent or temporary, pharmacological reversibility testing is used. The test is performed by repeating spirometry after inhalation of a rapid onset β2-agonist such as salbutamol [25,28]. The test is considered positive if there are FEV_1_ increases of 200 mL or FEV_1_ or FVC increases of 12% from the basal value [29]. A history of wheezing, cough, and chest tightness could be suggestive of the disorder, especially when there is a positive family history of asthma.

### 2.5. Ethical Considerations

The protocol was approved by the local Ethics Committee (Comitato di Bioetica, Azienda Ospedaliero-Universitaria di Sassari, Italy) (Protocol no. 3004/CE, 2016).

### 2.6. Statistical Analysis

Distributions of age, sex, socioeconomic status (SES), body mass index (BMI), and smoking habit were descriptively compared between cases and controls. More specifically, age was recoded into a binary variable by splitting at the age of 60 years. Socioeconomic status was estimated using current or past occupation and divided into four categories, from class I (the highest) to class IV (the lowest), as previously reported [30], and expressed as a binary variable: (i) high SES (classes I and II) and (ii) low SES (classes III and IV). BMI was calculated using the formula of weight (kg)/height (m^2^), and overweight/obesity was defined as BMI > 25 kg/m^2^. In relation to smoking habits, patients were stratified as never or current/former smokers. Patients with total and partial G6PD deficiency were pooled together.

Univariate and multivariable logistic regression was used to examine the association of asthma with G6PD status by calculating odds ratios (ORs) and their 95% confidence intervals (CI) using the Wald formula: 95% CI = OR^1±(β/SE)^. The main effect of variables was assessed first by entering the covariates one by one and then simultaneously (the adjusted model). In addition, patients with asthma were stratified according to the GINA classification, based on asthma symptoms and therapy, in order to ascertain the frequency of G6PD deficiency in each subgroup (intermittent, mild persistent, moderate persistent, and severe persistent asthma).

All statistical analyses were carried out using SPSS statistical software (version 22.0, Chicago, IL, USA), and two-sided *p* values lower than 0.05 were considered statistically significant.

## 3. Results

A total of 11,829 clinical records of patients (7323 females, 61.9%) who underwent a medical visit were available for the analysis. Descriptive statistics of the studied population are reported in Table 1.

The prevalence of asthma among study participants was 3.8%, according to a previous report [24]. Asthmatic patients were significantly younger than non-asthmatics (asthma: 49.1 ± 17.1 vs. non-asthmatics: 55.2 ± 18.0 years; *p* < 0.0001). As expected, the prevalence of asthma was greater among females compared with males (4.3 vs. 3.1, *p* = 0.001).

Individuals with a low SES were fewer in the asthmatic group (3.3% vs. 4.7%, *p* < 0.0001). Overweight/obesity (≥25 kg/m^2^) was more common among asthmatics, although this difference did not reach statistical significance (Table 1). The proportion of current or former smokers was significantly greater among asthmatics (4.8% vs. 3.5%, *p* = 0.003). Based on the laboratory data retrieved from the participants’ clinical records, 1319 patients (11.2%) were partially or totally deficient in G6PD, with greater frequency among females (13.4%) than among males (7.7%), in line with the sex-linked trait. Interestingly, the proportion of asthma sufferers was higher among G6PD-deficient patients than among patients with normal enzyme activity (5.8% vs. 3.6%, *p* < 0.0001) in both males (6.4%) and females (4.3%).

**Figure 1:**
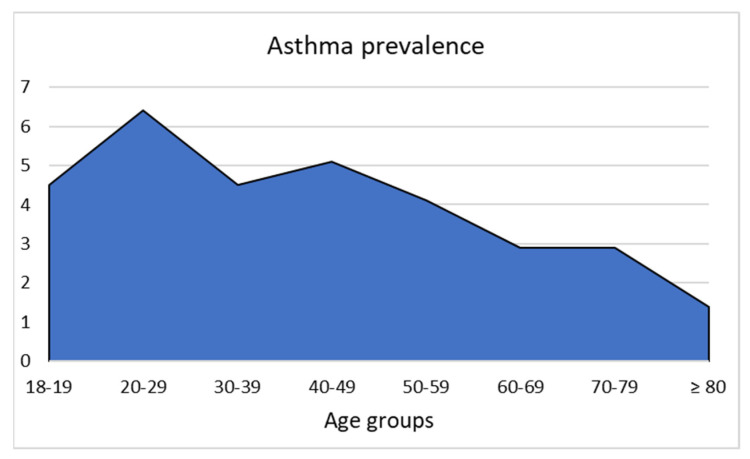
Asthma prevalence among the study participants, stratified by age groups.

The proportion of asthma decreased in patients in relation to age, as shown in Figure 1.

In Table 2, the risk of asthma occurrence is listed according to sex and age in G6PD-normal and -deficient patients.

The unadjusted risk of asthma was significantly higher than the unity among G6PD-deficient patients (1.69, 95% CI 1.31–2.17).

**Figure 2:**
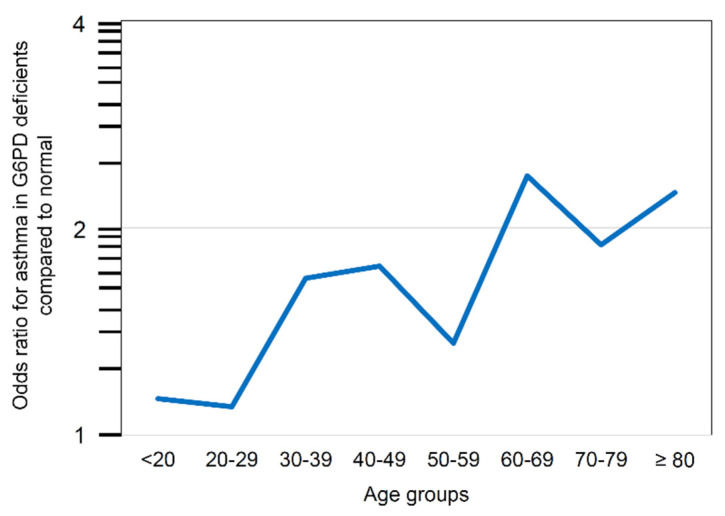
Asthma risk in G6PD-deficient patients compared to non-deficient patients according to age decades.

The increased risk of asthma among G6PD-deficient subjects was observed in both sexes, although statistical significance was reached in only the female subgroup. More specifically, an increasing trend was observed across the various age groups; the trend reached significance in the 60–79 years group (ANOVA, *p* < 0.0001) (Figure 2).

Remarkably, although the absolute risk of asthma was inversely related to age, in subjects with G6PD deficiency compared to normal subjects, the risk of asthma increased in an age-dependent manner (Figure 2) and reached a maximum after age 60 (OR 2.21, 95% CI 1.43–3.40) (Table 2).

Table 3 reports the univariate and multivariate logistic regression analysis for the association of G6PD status with asthma, adjusting for covariates. G6PD deficiency significantly increased the risk in the unadjusted model (1.69, 95% CI 1.31–2.17), remaining significant after adjusting for sex, age, SES, BMI, and smoking (1.63, 95% CI 1.27–2.10).

Table 4 reports the G6PD status according to the severity of the asthma phenotype. Compared with study participants without asthma, there was a trend to higher frequencies of G6PD deficiency among participants with the highest severity asthma levels, which was statistically significant for moderate persistent asthma.

Since steroids can inhibit G6PD activity, at least in animal models [31], there is a theoretical possibility that asthmatic patients may have an enzyme deficiency due to therapy. However, as can be seen in Table 4, the frequency of G6PD deficiency in patients with the most severe form of asthma, exposed to systemic steroids, was similar to that of the normal population.

## 4. Discussion

In the present study, conducted in a cohort of subjects referred to the Department of Medicine of Sassari, Italy, a significant association was found between inherited G6PD deficiency and the risk of asthma. This association, first reported over three decades ago [17,18], for many years, did not attract enough interest from investigators for two probable main reasons. First, the populations with the highest prevalence of G6PD deficiency live mostly in low-resource countries and, therefore, are less exposed to stressful environmental factors, such as pollution [32]. A second reason may be that until very recently, the enzyme was considered crucial only for red blood cells, which depend entirely on PPP for the maintenance of reducing capacity, while other cell types possess alternative sources of NADPH [33]. In reality, the expression of the enzyme is high in leukocytes and other immune cells [34], and, recently, G6PD deficiency has been found to be involved in several disorders beyond blood diseases, including sepsis [35], neonatal hyperbilirubinemia [36], and cardiovascular disease [37], especially in the elderly [38].

Among Sardinians, there is a prevalence of about 10–12% [22], essentially determined by a founder mutation (G6PD *Med*, S188F) in more than 95% of deficient subjects. The residual activity of G6PD *Med* is less than 10% in hemizygous males and homozygous females and between 10% and 50% in heterozygous females. Therefore, this population is an ideal model to test the hypothesis of the G6PD/asthma association with an adequate degree of statistical power.

The results obtained in this study provide compelling evidence that subjects with inherited G6PD deficiency are prone to develop asthma more frequently than subjects with normal enzyme activity, regardless of sex, SES, smoking habit, and excess weight. The increased risk was observed especially in older subjects, reaching statistical significance in the 60–79 years age group. The association probably persists in later ages as well, but the small number of older asthmatics in the examined sample cohort did not allow this hypothesis to be tested with sufficient statistical power. This finding suggests that G6PD-deficient individuals may experience progressively impaired antioxidant defense, putting them at increased risk to develop asthma in the presence of predisposing triggers, including aging. The same association may exist in children, although, in this study, it was not evaluated.

The main pathogenetic mechanism of asthma is due to chronic inflammation, largely driven by the innate immune response. Reactive oxygen species (ROS) are powerful mediators of the inflammatory response, and the increased production or decreased neutralization level of ROS plays an important role in the pathogenesis of asthma.

In a study aimed at identifying differentially expressed proteins in the serum of children with and without asthma, G6PD was found to be significantly downregulated in asthmatic children [39], suggesting that enzyme deficiency may increase ROS, triggering kinase pathways that are able to facilitate viral replication and, in turn, aggravating airways injury. An additional study performed on a pediatric cohort using the microarray profiling technique identified the G6PD gene among the first three differentially expressed genes out of the 274 genes associated with asthma [40]. G6PD deficiency could increase the risk of infections, including those of the respiratory tract [41], that are implicated in the etiology of asthma. It is known that in vitro cells with G6PD knockdown are more susceptible to viral infections [42], and during the recent COVID-19 pandemic, G6PD deficiency was seen as an aggravating factor in the clinical picture and prognosis [37]. It can, therefore, be hypothesized that children with G6PD deficiency experience viral and/or bacterial infections more frequently, paving the road for subsequent hyperreactivity.

In addition to infections, a key role in the pathogenesis of asthma is played by oxidative stress; this is supported by experimental and clinical studies, for example, those focused on exposure to strong oxidants such as ozone [43,44]. G6PD activity increases following intravenous administration of ozone in humans [45,46] as well as in alveolar macrophages in mouse models exposed to ozone [47]. Interestingly, Varghese et al. demonstrated that enzyme deficiency influences metabolic fluxes and pulmonary hypertension in a mouse line [48].

**Figure 3:**
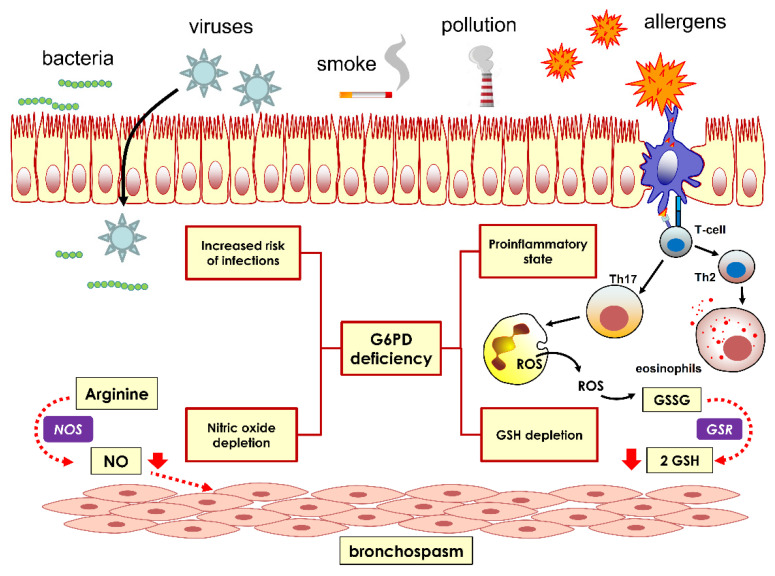
The putative role of G6PD deficiency in the pathogenesis of asthma. Nitric oxide (NO) production relaxes the smooth muscle of airways and vessels, inducing bronchodilation and vasodilation. G6PD deficiency induces bronchoconstriction by lowering NO generation by nitric oxide synthase (NOS). In addition, G6PD deficiency causes reduced glutathione (GSH) depletion, thus decreasing antioxidant defense and increasing the generation of free radicals.

A major function of G6PD is to regenerate the reduced thiol GSH consumed during the neutralization of ROS [49]. In experimental asthma models, the GSH is significantly decreased compared to controls, supporting the pivotal role of intracellular antioxidative mechanisms in this disorder [50]. Additionally, in human asthma, the level of reduced GSH is significantly increased, indicating a repairing function [51]. Thus, it is reasonable to speculate that G6PD-deficient subjects may have insufficient production of NADPH to maintain adequate GSH stores in asthma patients (Figure 3).

Furthermore, G6PD deficiency causes the depletion of nitric oxide (NO) [52]. This compound, being both a major signaling molecule and a free radical, has a dual effect: at low levels, it has mainly an airway-muscle-relaxing role, and, at high levels, it activates proinflammatory mechanisms; therefore, in asthma, the effect of NO depletion is complex, and, in severe G6PD, a deficiency may be beneficial as well as adverse. It can be conjectured that the chronic depletion of NO, resulting from G6PD deficiency, may affect the basal bronchodilator tone sustained by this molecule [53]. More importantly, NO depletion manifests its effect, especially in the elderly, where there is a progressive impairment of antioxidant mechanisms [54]; perhaps this may partly explain why in our study, the magnitude of the association of G6PD deficiency with asthma was higher in patients after age 60 (Figure 3).

Finally, G6PD deficiency is associated with a proinflammatory state or the exacerbation of a proinflammatory state [21]. Recently, an increasing number of studies have reported that G6PD deficiency alters specific cytokine pathways involved in several disorders. For example, an in vitro model based on human hepatocarcinoma HepG2 cells, in which G6PD was inactivated by siRNA, revealed an increased production of neutrophil chemoattractant interleukin-8 (IL-8), which was mirrored by an enhanced production of reactive oxygen species [55]. At the molecular level, the mechanism has been investigated in detail and involves the activation of the NF-κB signaling pathway. Increased serum levels of IL-8, as well IL-4, IL-5, and IL-9 have been reported in asthma hyperreactivity following Th2 lymphocyte release [56]. Therefore, G6PD-deficient subjects may experience over-expression of IL-8 and increased chemotaxis of eosinophils and other inflammatory cells, which are found in abundance in sputum or bronchoalveolar lavage fluid. Moreover, since TGF-β secretion was found to be increased in G6PD-deficient macrophages [57], this can be an aggravating factor of the inflammatory state (Figure 3).

The association of asthma with the G6PD gene, which maps on the X chromosome (OMIM 305900), could theoretically be due to a linkage with a predisposing gene located near the G6PD locus. The most interesting candidate is probably the cysteinyl leukotriene receptor 1 (CYSLTR1) gene, located on chromosome Xq21.1 [58], which has been implicated in several allergic disorders, including asthma [59]. However, the relative distance between the two loci does not totally exclude the possibility of recombination events, making this hypothesis weak and implicitly strengthening a direct pathogenetic role of the G6PD gene itself.

Some limitations of this study need to be mentioned. First, being a retrospective study, we cannot exclude that potential confounders may be differently distributed between cases and controls. However, given the large number of participants, we are confident that the extent of such a bias would be minimized. An additional limitation of our study may be the lack of molecular genotyping for patients with biochemically identified G6PD deficiency, precluding the possibility, in the case of females, of distinguishing homozygotes and heterozygotes and, therefore, testing the association of asthma with total or partial G6PD deficiency separately. However, from a clinical standpoint, the lack of a more detailed analysis would not have changed the overall findings.

## 5. Conclusions

In conclusion, in our study, we found that G6PD deficiency was associated with an increased risk of asthma, and this risk remained significant after adjusting for well-known traditional variables such as female gender, overweight/obesity, and high SES. These findings suggest that G6PD should be assessed in patients with asthma for better risk stratification.

## Figures and Tables

**Table 1:** Characteristics of 11,829 study participants with and without asthma.

Covariates	Asthma (Cases)	No Asthma (Controls)	*p*-Value
Age, *n* (%)			
<60	322 (4.8)	6352 (95.2)	
≥60	133 (2.6)	5022 (97.4)	<0.0001
Sex, *n* (%)			
Female	315 (4.3)	7008 (95.7)	
Male	140 (3.1)	4366 (96.9)	0.001
SES ^1^, *n* (%)			
Low	230 (3.3)	6807 (96.7)	
High	225 (4.7)	4567 (95.3)	<0.0001
BMI ^2^, kg/m^2^			
<25	211 (3.6)	5652 (96.4)	
≥25	244 (4.1)	5722 (95.9)	0.166
Smoke			
No	316 (3.5)	8602 (96.5)	
Yes	139 (4.8)	2772 (95.2)	0.003
G6PD ^3^ status			
Normal	378 (3.6)	10,132 (96.4)	
Deficiency	77 (5.8)	1242 (94.2)	<0.0001

^1^ Socioeconomic status; ^2^ body mass index; ^3^ glucose-6-phosphate dehydrogenase.

**Table 2:** Risk of asthma according to sex and age in G6PD-normal and -deficient individuals.

	Cases (Asthma)		Controls (No Asthma)		OR ^§^ (95% CI ^†^)
	G6PD ^#^ Normal	G6PD Deficient	G6PD Normal	G6PD Deficient	
Sex, *n* (%)					
Female	253	62 (19.6)	6097	911 (12.9)	1.64 (1.23–2.18) **
Male	124	16 (11.4)	4035	331 (7.6)	1.57 (0.92–2.68)
Age, *n* (%)					
<60	271	51 (15.8)	5629	723 (11.4)	1.47 (1.08–2.00) *
≥60	106	27 (20.3)	4503	519 (10.3)	2.21 (1.43–3.40) **
Total patients	377	78 (17.1)	10,132	1242 (10.9)	1.69 (1.31–2.17) **

^§^ OR = odds ratio; ^†^ CI = confidence interval; ^#^ G6PD = glucose-6-phosphate dehydrogenase; * *p* < 0.05, ** *p* < 0.01.

**Table 3:** Logistic regression analysis for G6PD status and other variables potentially associated with the risk of asthma.

Covariates	Unadjusted OR ^‡^s and 95% CI	Adjusted ORs and 95% CI
G6PD ^#^ status		
Normal	Ref	Ref
Deficiency	1.69 (1.31–2.17) **	1.63 (1.27–2.10) **
Age, yrs		
<60	Ref	Ref
≥60	0.52 (0.42–0.64) **	0.49 (0.39–0.61) **
Sex		
Male	Ref	Ref
Female	1.40 (1.14–1.72) **	1.66 (1.34–2.06) **
SES ^§^		
Low	Ref.	Ref.
High	1.46 (1.21–1.76) **	1.40 (1.16–1.70) **
BMI, kg/m^2^		
<30	Ref.	Ref.
≥30	1.14 (0.94–1.38)	1.56 (1.27–1.92) **
Smoking		
No	Ref.	Ref.
Yes	1.36 (1.11–1.67) **	1.44 (1.17–1.77) **

^#^ G6PD = glucose-6-phosphate dehydrogenase, ^§^ SES = socioeconomic status, ^‡^ OR = odds ratio; ** *p* < 0.01.

**Table 4:** The severity of asthma based on the Global Initiative for Asthma (GINA) classification according to glucose-6-phosphate dehydrogenase (G6PD) status.

Severity of Asthma According to GINA ^#^ Guidelines	Drugs Used to Treat Asthma	G6PD-Normal No. (%)	G6PD-Deficient No. (%)
Intermittent asthma	Low dose ICS ^§^-formoterol as needed, with rapid onset LABA ^¶^ as needed, or low-dose ICS whenever SABA ^$^ used	206 (85.5)	35 (14.5)
Mild persistent asthma	Daily low dose ICS with SABA or low dose ICS-formoterol as needed, or low-dose ICS plus SABA ^$^ concomitantly as needed, or LTRA^‡^ daily and SABA ^$^ as needed	74 (84.1)	14 (15.9)
Moderate persistent asthma	Low dose ICS-LABA as maintenance and reliever therapy, or low-dose ICS plus LTRA daily, +/− SABA as needed	84 (76.4)	26 (23.6) **
Severe persistent asthma	Medium or high-dose ICS-LABA daily and SABA as needed, or high dose of ICS plus tiotropium, or LTRA +/− short course of oral glucocorticoids +/− add-on therapy (e.g., tiotropium, zileuton, anti-IgE, anti-IL-5, anti-IL-5R, anti-IL-4R), or oral glucocorticoids, or addiction of biologics	14 (87.5)	2 (12.5)

^#^ GINA, Global Initiative for Asthma; ^§^ ICS, inhaled corticosteroid; ^¶^ LABA, long-acting beta agonists; ^$^ SABA, short-acting beta agonists ^‡^ LTRA, leukotriene receptor antagonists; ** *p* < 0.01.

## Data Availability

The data presented in this study are available on request from the corresponding author.

## References

[1] The Global Asthma Report. http://globalasthmareport.org/resources/Global_Asthma_Report_2018.pdf.

[2] 2D’Amato G., Holgate S.T., Pawankar R., Ledford D.K., Cecchi L., Al-Ahmad M., Al-Enezi F., Al-Muhsen S., Ansotegui I., Baena-Cagnani C.E. (2015). Meteorological Conditions, Climate Change, New Emerging Factors, and Asthma and Related Allergic Disorders. A Statement of the World Allergy Organization. World Allergy Organ. J..

[3] To T., Stanojevic S., Moores G., Gershon A.S., Bateman E.D., Cruz A.A., Boulet L.P. (2012). Global Asthma Prevalence in Adults: Findings from the Cross-Sectional World Health Survey. BMC Public Health.

[4] Ridolo E., Incorvaia C., Martignago I., Caminati M., Canonica G.W., Senna G. (2019). Sex in Respiratory and Skin Allergies. Clin. Rev. Allergy Immunol..

[5] Holgate S.T. (2008). Pathogenesis of Asthma. Clin. Exp. Allergy.

[6] Deschamps K., Cromlish W., Weicker S., Lamontagne S., Huszar S.L., Gauthier J.Y., Mudgett J.S., Guimond A., Romand R., Frossard N. (2011). Genetic and Pharmacological Evaluation of Cathepsin s in a Mouse Model of Asthma. Am. J. Respir. Cell Mol. Biol..

[7] Smit J.J., Lukacs N.W. (2006). A Closer Look at Chemokines and Their Role in Asthmatic Responses. Eur. J. Pharmacol..

[8] Chen M., Sun Y., Wu Y. (2020). Lower Circulating Zinc and Selenium Levels Are Associated with an Increased Risk of Asthma: Evidence from a Meta-Analysis. Public Health Nutr..

[9] Cook-Mills J., Gebretsadik T., Abdala-Valencia H., Green J., Larkin E.K., Dupont W.D., Shu X.O., Gross M., Bai C., Gao Y.T. (2016). Interaction of Vitamin E Isoforms on Asthma and Allergic Airway Disease. Thorax.

[10] Wang T., Li J., Liang Y., Han W., Tang J., Cheng G., Zheng Y. (2021). Joint Effects of Carbon Black Exposure and Dietary Antioxidant Vitamin Intake on Small Airway Dysfunction. Front. Nutr..

[11] Carroll K.N., Gebretsadik T., Griffin M.R., Dupont W.D., Mitchel E.F., Wu P., Enriquez R., Hartert T.V. (2007). Maternal Asthma and Maternal Smoking Are Associated with Increased Risk of Bronchiolitis during Infancy. Pediatrics.

[12] Lu M., Litonjua A.A., O’Connor G.T., Zeiger R.S., Bacharier L., Schatz M., Carey V.J., Weiss S.T., Mirzakhani H. (2021). Effect of Early and Late Prenatal Vitamin D and Maternal Asthma Status on Offspring Asthma or Recurrent Wheeze. J. Allergy Clin. Immunol..

[13] Liew Z., Nohr E.A., Morgen C.S., Ernst A., Li J., Sorensen T.I.A., Olsen J. (2019). Prenatal Exposure to Acetaminophen and Overweight in Childhood. Obesity.

[14] Lai T., Wu M., Liu J., Luo M., He L., Wang X., Wu B., Ying S., Chen Z., Li W. (2018). Acid-Suppressive Drug Use during Pregnancy and the Risk of Childhood Asthma: A Meta-analysis. Pediatrics.

[15] Kankaanranta H., Kauppi P., Tuomisto L.E., Ilmarinen P. (2016). Emerging Comorbidities in Adult Asthma: Risks, Clinical Associations, and Mechanisms. Mediat. Inflamm..

[16] Thacher J.D., Gehring U., Gruzieva O., Standl M., Pershagen G., Bauer C.P., Berdel D., Keller T., Koletzko S., Koppelman G.H. (2018). Maternal Smoking during Pregnancy and Early Childhood and Development of Asthma and Rhinoconjunctivitis - a MeDALL Project. Environ. Health Perspect..

[17] Greene L.S. (1995). Asthma and Oxidant Stress: Nutritional, Environmental, and Genetic Risk Factors. J. Am. Coll. Nutr..

[18] Tyran W., Wrzyszcz M. (1992). Activity of Glucose-6-Phosphate Dehydrogenase in Erythrocytes in Patients with Atopic Asthma and Allergic Rhinitis. Pneumonol. Alergol. Pol..

[19] Luzzatto L. (1974). Genetic Heterogeneity and Pathophysiology of G6PD Deficiency. Br. J. Haematol..

[20] Luzzatto L., Ally M., Notaro R. (2020). Glucose-6-Phosphate Dehydrogenase Deficiency. Blood.

[21] Parsanathan R., Jain S.K. (2021). G6PD Deficiency Shifts Polarization of Monocytes/Macrophages towards a Proinflammatory and Profibrotic Phenotype. Cell Mol. Immunol..

[22] Fiorelli G., Meloni T., Palomba V., Manoussakis C., Villa S., Cappellini M.D. (1990). Gene Frequency of Glucose-6-Phosphate Dehydrogenase (G6PD) Polymorphic Variants in Sardinia. Gene. Geogr..

[23] de Marco R., Cappa V., Accordini S., Rava M., Antonicelli L., Bortolami O., Braggion M., Bugiani M., Casali L., Cazzoletti L. (2012). Trends in the Prevalence of Asthma and Allergic Rhinitis in Italy between 1991 and 2010. Eur. Respir. J..

[24] Ferrante G., Baldissera S., Campostrini S. (2017). Epidemiology of Chronic Respiratory Diseases and Associated Factors in the Adult Italian Population. Eur. J. Public Health.

[25] Global Initiative for Asthma (2019). Asthma Management and Prevention for Adults and Children Older Than 5 Years. A Pocket Guide for Health Professionals.

[26] Dore M.P., Marras G., Rocchi C., Soro S., Pes G.M. (2016). G6PD Deficiency Does Not Enhance Susceptibility for Acquiring Helicobacter pylori Infection in Sardinian Patients. PLoS ONE.

[27] Mosca A., Paderi M., Sanna A., Paleari R., Cao A., Galanello R. (1990). Preliminary Experience with the Differential pH Technique for Glucose-6-Phosphate Dehydrogenase (G6PD) Measurement in Whole Blood: Application to an Area with High Prevalence of Thalassaemia and G6PD Deficiency. Haematologica.

[28] Chung K.F., Wenzel S.E., Brozek J.L., Bush A., Castro M., Sterk P.J., Adcock I.M., Bateman E.D., Bel E.H., Bleecker E.R. (2014). International ERS/ATS Guidelines on Definition, Evaluation and Treatment of Severe Asthma. Eur. Respir. J..

[29] Quanjer P.H., Ruppel G.L., Langhammer A., Krishna A., Mertens F., Johannessen A., Menezes A.M.B., Wehrmeister F.C., Perez-Padilla R., Swanney M.P. (2017). Bronchodilator Response in FVC Is Larger and More Relevant Than in FEV1 in Severe Airflow Obstruction. Chest.

[30] Pes G.M., Ganau A., Tognotti E., Errigo A., Rocchi C., Dore M.P. (2018). The Association of Adult Height with the Risk of Cardiovascular Disease and Cancer in the Population of Sardinia. PLoS ONE.

[31] Ozmen I. (2005). Evaluation of Effect of Some Corticosteroids on Glucose-6-Phosphate Dehydrogenase and Comparative Study of Antioxidant enzyme Activities. J. Enzyme Inhib. Med. Chem..

[32] de Marco R., Poli A., Ferrari M., Accordini S., Giammanco G., Bugiani M., Villani S., Ponzio M., Bono R., Carrozzi L. (2002). The Impact of Climate and Traffic-Related NO2 on the Prevalence of Asthma and Allergic Rhinitis in Italy. Clin. Exp. Allergy.

[33] Mehta A., Mason P.J., Vulliamy T.J. (2000). Glucose-6-Phosphate Dehydrogenase Deficiency. Baillieres Best Pr. Res. Clin. Haematol..

[34] Battistuzzi G., D’Urso M., Toniolo D., Persico G.M., Luzzatto L. (1985). Tissue-Specific Levels of Human Glucose-6-Phosphate Dehydrogenase Correlate with Methylation of Specific Sites at the 3′ End of the Gene. Proc. Natl. Acad. Sci. USA.

[35] Spolarics Z., Siddiqi M., Siegel J.H., Garcia Z.C., Stein D.S., Ong H., Livingston D.H., Denny T., Deitch E.A. (2001). Increased Incidence of Sepsis and Altered Monocyte Functions in Severely Injured Type A- Glucose-6-Phosphate Dehydrogenase-Deficient African American Trauma Patients. Crit. Care Med..

[36] Atay E., Bozaykut A., Ipek I.O. (2006). Glucose-6-Phosphate Dehydrogenase Deficiency in Neonatal Indirect Hyperbilirubinemia. J. Trop. Pediatr..

[37] Dore M.P., Parodi G., Portoghese M., Pes G.M. (2021). The Controversial Role of Glucose-6-Phosphate Dehydrogenase Deficiency on Cardiovascular Disease: A Narrative Review. Oxid. Med. Cell Longev..

[38] Dore M.P., Portoghese M., Pes G.M. (2021). The Elderly with Glucose-6-Phosphate Dehydrogenase Deficiency are More Susceptible to Cardiovascular Disease. J. Atheroscler. Thromb..

[39] Li M., Wu M., Qin Y., Liu H., Tu C., Shen B., Xu X., Chen H. (2020). Differentially Expressed Serum Proteins in Children with or without Asthma as Determined Using Isobaric Tags for Relative and Absolute Quantitation Proteomics. PeerJ.

[40] Chen Y., Qiao J. (2015). Protein-Protein Interaction Network Analysis and Identifying Regulation microRNAs in Asthmatic Children. Allergol. Immunopathol..

[41] Beutler E. (1994). G6PD Deficiency. Blood.

[42] Wu Y.H., Chiu D.T., Lin H.R., Tang H.Y., Cheng M.L., Ho H.Y. (2015). Glucose-6-Phosphate Dehydrogenase Enhances Antiviral Response through Downregulation of NADPH Sensor HSCARG and Upregulation of NF-kappaB Signaling. Viruses.

[43] Fanucchi M.V., Plopper C.G., Evans M.J., Hyde D.M., Van Winkle L.S., Gershwin L.J., Schelegle E.S. (2006). Cyclic Exposure to Ozone Alters Distal Airway Development in Infant Rhesus Monkeys. Am. J. Physiol. Lung Cell Mol. Physiol..

[44] Malig B.J., Pearson D.L., Chang Y.B., Broadwin R., Basu R., Green R.S., Ostro B. (2016). A Time-Stratified Case-Crossover Study of Ambient Ozone Exposure and Emergency Department Visits for Specific Respiratory Diagnoses in California (2005–2008). Env. Health Perspect..

[45] Hernandez F., Menendez S., Wong R. (1995). Decrease of Blood Cholesterol and Stimulation of Antioxidative Response in Cardiopathy Patients Treated with Endovenous Ozone Therapy. Free Radic. Biol. Med..

[46] Linn W.S., Buckley R.D., Spier C.E., Blessey R.L., Jones M.P., Fischer D.A., Hackney J.D. (1978). Health Effects of Ozone Exposure in Asthmatics. Am. Rev. Respir. Dis..

[47] Mochitate K., Miura T. (1989). Metabolic Enhancement and Increase of Alveolar Macrophages Induced by Ozone. Environ. Res..

[48] Varghese M.V., James J., Rafikova O., Rafikov R. (2021). Glucose-6-Phosphate Dehydrogenase Deficiency Contributes to Metabolic Abnormality and Pulmonary Hypertension. Am. J. Physiol. Lung Cell Mol. Physiol..

[49] Kurata M., Suzuki M. (1994). Glutathione Regeneration in Calcium-Loaded Erythrocytes: A Possible Relationship among Calcium Accumulation, ATP Decrement and Oxidative Damage. Comp. Biochem. Physiol. B Biochem. Mol. Biol..

[50] Wang Q., Li A., Zheng Y., Zhang S., Wang P. (2020). Glutathione ethyl Ester Supplementation Prevents Airway Hyper-Responsiveness in Mice. Ann. Transl. Med..

[51] Ahmad A., Shameem M., Husain Q. (2012). Relation of Oxidant-Antioxidant Imbalance with Disease Progression in Patients with Asthma. Ann. Thorac. Med..

[52] Parsanathan R., Jain S.K. (2019). Glucose-6-Phosphate Dehydrogenase Deficiency Increases Cell Adhesion Molecules and Activates Human Monocyte-Endothelial Cell Adhesion: Protective Role of l-Cysteine. Arch. Biochem. Biophys..

[53] Prado C.M., Martins M.A., Tiberio I.F. (2011). Nitric Oxide in Asthma Physiopathology. ISRN Allergy.

[54] Pourbagher-Shahri A.M., Farkhondeh T., Talebi M., Kopustinskiene D.M., Samarghandian S., Bernatoniene J. (2021). An Overview of NO Signaling Pathways in Aging. Molecules.

[55] Yang H.C., Cheng M.L., Hua Y.S., Wu Y.H., Lin H.R., Liu H.Y., Ho H.Y., Chiu D.T. (2015). Glucose 6-Phosphate Dehydrogenase Knockdown Enhances IL-8 Expression in HepG2 Cells via Oxidative Stress and NF-kappaB Signaling Pathway. J. Inflamm..

[56] Matsuda S., Kato M., Koike T., Kama Y., Suzuki K., Enseki M., Tabata H., Hirai K., Yamada Y., Mochizuki H. (2020). Differences in Virus Detection and Cytokine Profiles between First Wheeze and Childhood Asthma. Tokai J. Exp. Clin. Med..

[57] Sanna F., Bonatesta R.R., Frongia B., Uda S., Banni S., Melis M.P., Collu M., Madeddu C., Serpe R., Puddu S. (2007). Production of Inflammatory Molecules in Peripheral Blood Mononuclear Cells from Severely Glucose-6-Phosphate Dehydrogenase-Deficient Subjects. J. Vasc. Res..

[58] Lynch K.R., O’Neill G.P., Liu Q., Im D.S., Sawyer N., Metters K.M., Coulombe N., Abramovitz M., Figueroa D.J., Zeng Z. (1999). Characterization of the Human Cysteinyl Leukotriene CysLT1 Receptor. Nature.

[59] Duroudier N.P., Strachan D.P., Blakey J.D., Hall I.P. (2009). Association of the Cysteinyl Leukotriene Receptor 1 Gene with Atopy in the British 1958 Birth Cohort. J. Allergy Clin. Immunol..

